# Effects of *Cdh23* single nucleotide substitutions on age-related hearing loss in C57BL/6 and 129S1/Sv mice and comparisons with congenic strains

**DOI:** 10.1038/srep44450

**Published:** 2017-03-13

**Authors:** Kenneth R. Johnson, Cong Tian, Leona H. Gagnon, Haiyan Jiang, Dalian Ding, Richard Salvi

**Affiliations:** 1 The Jackson Laboratory, Bar Harbor, Maine 04609, USA; 2 Center for Hearing and Deafness, University at Buffalo, Buffalo, NY 14214, USA

## Abstract

A single nucleotide variant (SNV) of the cadherin 23 gene (*Cdh23*^*c.753A*^), common to many inbred mouse strains, accelerates age-related hearing loss (AHL) and can worsen auditory phenotypes of other mutations. We used homologous recombination in C57BL/6 NJ (B6N) and 129S1/SvImJ (129S1) embryonic stem cells to engineer mouse strains with reciprocal single base pair substitutions (B6-*Cdh23*^*c.753A*>*G*^ and 129S1-*Cdh23*^*c.753G*>*A*^). We compared ABR thresholds and cochlear pathologies of these SNV mice with those of congenic (B6.129S1-*Cdh23*^*Ahl*+^ and 129S1.B6-*Cdh23*^*ahl*^) and parental (B6N and 129S1) strain mice. Results verified the protective effect of the *Cdh23*^*c.753G*^ allele, which prevented high frequency hearing loss in B6 mice to at least 18 months of age, and the AHL-inducing effect of the *Cdh23*^*c.753A*^ allele, which worsened hearing loss in 129S1 mice. ABR thresholds differed between 129S-*Cdh23*^*c.753A*^ SNV and 129S1.B6-*Cdh23*^*ahl*^ congenic mice, and a linkage backcross involving these strains localized a Chr 10 QTL contributing to the difference. These results illustrate the large effects that strain background and congenic regions have on the hearing loss associated with *Cdh23*^*c.753*^alleles. Importantly, the B6-*Cdh23*^*c.753G*^strain can be used to eliminate the confounding influence of the *Cdh23*^*c.753A*^variant in hearing studies of B6 mice and mutant mice on the B6 background.

Age-related hearing loss (AHL) is the most prevalent sensory impairment in elderly individuals and is becoming a major health concern with world population aging^1^. AHL has been shown to have a significant genetic component^2^,^3^; however because of its complex nature and late onset, causative gene variants influencing AHL susceptibility have been difficult to identify in human populations^4^. Mice serve as valuable model systems for studying human hearing loss because their auditory system is similar to that of humans and because genetic and environmental variation can be manipulated and controlled in mice^5^,^6^,^7^. Genetically homogeneous inbred strains of mice vary widely in onset times and progression of hearing loss^8^ making them highly amenable to genetic studies of AHL. The C57BL/6 (B6) strain, widely used in many research fields, has long been studied as a model for AHL^9^,^10^,^11^.

A major genetic factor contributing to AHL in B6 mice was mapped to a locus (named *ahl*) on Chr 10^12^ and later shown to be a common variant in many other inbred strains^13^. The coincident map position of *ahl* with cadherin 23 (*Cdh23*) and the correspondence of specific *Cdh23* alleles with hearing loss severity in multiple inbred strains provided evidence that a single nucleotide variant (SNV) of *Cdh23* underlies the *ahl* phenotype^14^. This SNV corresponds to SNP *rs257098870* and is at the 753rd nucleotide position from the ATG translation start site of *Cdh23* cDNA (c.753), which corresponds to the last nucleotide of exon 7 in GenBank sequence AF308939 and to the last nucleotide of exon 9 in Ref Seqs NM_023370 and NM_001252635. A G nucleotide at this site (*Cdh23*^*c.753G*^) results in normal exon splicing, whereas an A nucleotide (*Cdh23*^*c.753A*^) disrupts the canonical donor splice site sequence and causes in-frame exon skipping^14^. The *Cdh23*^*c.753G*^ allele is associated with resistance to AHL and is dominant to the recessive *Cdh23*^*c.753A*^ allele, which is associated with AHL susceptibility.

The analyses of congenic strains, including B6.CAST-*Cdh23*^*Ahl*+^/Kjn^12^ and B6.CBACa-*Cdh23*^*CBA/CaJ*^/Kjn^15^, provided evidence that CAST/EiJ- and CBA/CaJ-derived allelic substitutions at the *ahl* locus could alleviate the progression of hearing loss in B6 mice, but the protective effect could not be definitively attributed to the *Cdh23* gene because of possible contributions from linked genes in the congenic regions of these strains. Recently, *Cdh23*^*c.753A*>*G*^ single base pair substitutions created by CRISPR-Cas9 genome editing were shown to prevent AHL in B6 strain mice^16^ and to prevent hearing loss in *Ush1g*^*js*^ heterozygotes on the B6 strain background^17^, verifying the causative nature of the *Cdh23*^*c.753A*^ variant.

Here, we describe our analyses of C57BL/6NJ mice with a *Cdh23*^*c.753A*>*G*^ single nucleotide substitution and 129S1/SvImJ mice with the reciprocal *Cdh23*^*c.753G*>*A*^ substitution produced by means of the traditional targeting approach using homologous recombination in embryonic stem (ES) cells. These strains were chosen because of their widespread use and the availability of strain-matched ES cells and BAC clones. We compared, over an 18-month time course, the auditory phenotypes (hearing loss and cochlear pathology) of these single nucleotide variant mice with those of mice from their parental strains and with mice from corresponding congenic strains. Our results provide insight into the genetic and pathological mechanisms underlying progressive hearing loss in these two commonly used inbred strains of mice with potential implications for human genetic studies of age-related hearing loss.

## Results

**Figure 1:**
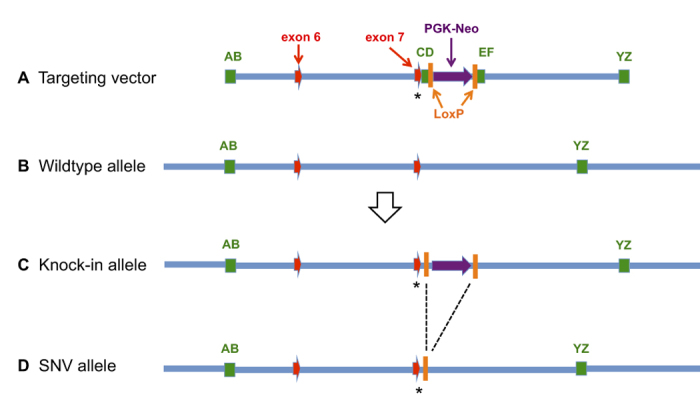
Recombineering strategy used to produce 129S-*Cdh23*^*c.753A*^ and B6-*Cdh23*^*c.753G*^ SNV mice. To produce B6N strain mice with the *Cdh23*^*c.753G*^ SNV, a targeting vector with DNA from a 129S1/Sv BAC clone was constructed for recombination with C57BL/6 N ES cells (JM8.A3). To produce 129S strain mice with the *Cdh23*^*c.753A*^ SNV, a targeting vector with DNA from a C57BL/6 J BAC clone was constructed for recombination with 129S1/SvImJ ES cells. (**A**) Targeting vector: The *Cdh23* targeted region (9,729 bp) was cloned into a linearized pBlight plasmid (4695 bp) by retrieval from a BAC clone with AB (300 bp) and YZ (320 bp) homology arms. PGK-Neo and LoxP sites then were inserted into the plasmid by retrieval from a synthesized DNA cassette (2320 bp) with CD (200 bp) and EF (203 bp) homology arms. The targeted *Cdh23*^*c.753*^ SNV (marked by asterisk) is separated from the cassette insertion site by 142 bp and from PGK-Neo by 178 bp. In this figure, the targeted *Cdh23*^*c.753*^ SNV is shown as the last nucleotide of exon 7 according to Genbank transcript AF308939, which is equivalent to exon 9 in other transcripts such as NM_023370. (**B**) Wildtype allele: The targeted *Cdh23* region of wildtype mouse genomic DNA showing positions of AB and YZ homology arms relative to exons 6 and 7. (**C**) Knock-in allele: After homologous recombination between the targeting vector and the wildtype *Cdh23* allele, ES cells were selected for the presence of the PGK-Neo cassette and the closely linked targeted SNV (asterisk). Restriction enzyme cleavage sites and genomic probes (5′ and 3′) were used to distinguish wildtype and knock-in alleles. (**D**) The PGK-Neo cassette was subsequently removed by Cre-Lox recombination to produce the final SNV allele.

**Figure 2:**
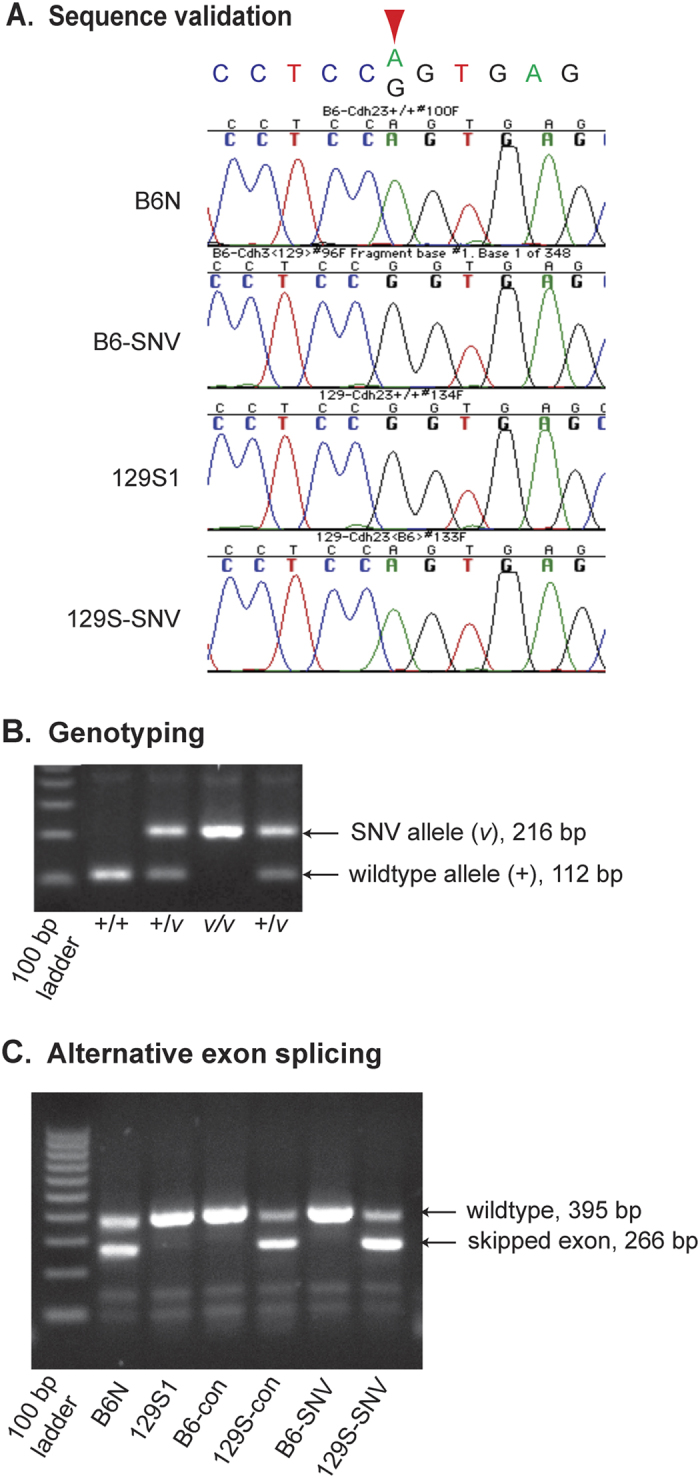
DNA sequence validation, PCR identification of targeted SNVs and assessment of exon skpping. (**A**) Sequence chromatograms of PCR amplified DNA surrounding the targeted *Cdh23*^*c.753*^ nucleotide (indicated by the red downward-pointing arrow) confirm that C57BL/6 NJ (B6N) and 129S-*Cdh23*^*c.753A*^ (129S-SNV) mice are homozygous for the *Cdh23* c.753 A nucleotide, while 129S1/SvImJ (129S1) and B6N-*Cdh23*^*c.753G*^ (B6N-SNV) mice are homozygous for the *Cdh23* c.753 G nucleotide. (**B**) Identification of *Cdh23* alleles with targeted SNVs by PCR amplification of the closely linked PGK-Neo insertion remnant. Primers flanking the PGK-Neo cassette insertion site were used to amplify PCR products that differ in size between the wildtype allele and the targeted SNV allele, which retains an intronic 104 bp remnant of the PGK-Neo cassette after Cre deletion. Because of its close proximity (178 bp) to the targeted SNV, the presence or absence of the PGK-Neo remnant can be used to distinguish the wildtype allele (+, 112 bp) from the targeted SNV allele (*v*, 216 bp). Lane 1, 100-bp DNA size ladder; lanes 2–5, genotypes of individual mice. (**C**) RT-PCR to evaluate the extent of exon skipping related to the *Cdh23*^*c.753A*^ variant. cDNA primers flanking the alternatively spliced exon of *Cdh23* (containing the c.753 nucleotide) were used to amplify alternatively spliced products. The PCR product size from the wild-type *Cdh23* transcript (395 bp) is larger than the PCR product from the alternatively spliced, in-frame transcript (266 bp), which lacks the 129 bp skipped exon. Lane 1, 100-bp DNA size ladder; lane 2, B6N; lane 3, 129S1; lane 4, B6.129S1-*Cdh23*^*Ahl*+^ congenic (B6-con); lane 5, 129S1.B6-*Cdh23*^*ahl*^ congenic (129S-con); lane 6, B6-*Cdh23*^*c.753G*^ SNV (B6-SNV); and lane 7, 129S-*Cdh23*^*c.753A*^SNV (129S-SNV). Lanes 2, 5, and 7 show alternatively spliced transcripts caused by the *Cdh23*^*c.753A*^ variant.

Full strain nomenclature and Jackson Laboratory stock numbers are given in Table 1 for the abbreviated strain designations used henceforth. 129S1 and B6 ES cells were used in a recombineering approach to create the 129S-*Cdh23*^*c.753A*^ and B6-*Cdh23*^*c.753G*^ SNV strains (Fig. 1), and the targeted single nucleotide substitutions in these strains were verified by DNA sequence analysis (Fig. 2A). A simple PCR method was developed to identify mice with the genetically engineered *Cdh23*^*c.753A/G*^ substitutions and distinguish their genotypes (Fig. 2B).

For comparisons with the SNV strain mice, we generated two corresponding congenic strains, B6.129S1-*Cdh23*^*Ahl*+^ and 129S1.B6-*Cdh23*^*ahl*^, by recurrent backcrossing and selection. The 44–48 Mb 129S1-derived Chr 10 congenic region of B6.129S1-*Cdh23*^*Ahl*+^ extends from a breakpoint between *rs3665690* (23.19 Mb position) and *rs13480546* (24.28 Mb position) to a breakpoint between *rs13480629* (67.62 Mb position) and *rs13480646* (71.25 Mb position). The 7–18 Mb B6J-derived Chr 10 congenic region of 129S1.B6-*Cdh23*^*ahl*^ extends from a breakpoint between *rs3661754* (44.97 Mb position) and *rs3696307* (53.74 Mb position) to a breakpoint between *rs257098870* (60.53 Mb position) and *rs29330969* (63.15 Mb position).

### Exon skipping is associated with the *Cdh23* ^*c.753A*^ variant

RNA extracted from eight cochleae (four mice) from each of the SNV and congenic strains and from the B6 and 129S1 parental strains were evaluated for *Cdh23* alternative exon splicing by RT-PCR (Fig. 2C). As shown previously by others^14^,^17^, exon skipping was specifically associated with the *Cdh23*^*c.753A*^ variant. Our results (Fig. 2) showed exon skipping in cochlear transcripts from B6N parental (lane 2), 129S1.B6-*Cdh23*^*ahl*^congenic (lane 5), and 129S-*Cdh23*^*c.753A*^ SNV (lane 7) strains, but not in 129S1 parental (lane 3), B6.129S1-*Cdh23*^*Ahl*+^ congenic (lane 4), and B6-*Cdh23*^*c.753G*^ SNV (lane 6) strains. For each of the three strains exhibiting exon skipping, we compared the image density of the gel band corresponding to the wildtype allele (larger, upper band) to the density of the band corresponding to the exon-skipped allele (smaller, lower band) using the ImageJ software program. From these gel band density ratios, we estimated that the wildtype allele comprises about 45% of the *Cdh23* transcripts in cochleae of B6 mice, about 24% in 129S1.B6-*Cdh23*^*ahl*^ congenic mice, and only about 18% in 129S-*Cdh23*^*c.753A*^ SNV mice.

### Hearing loss progression differs in B6N, B6.129S1-*Cdh23* ^*Ahl*+^, and *B6-Cdh23* ^*c.753G*^ mice

**Figure 3:**
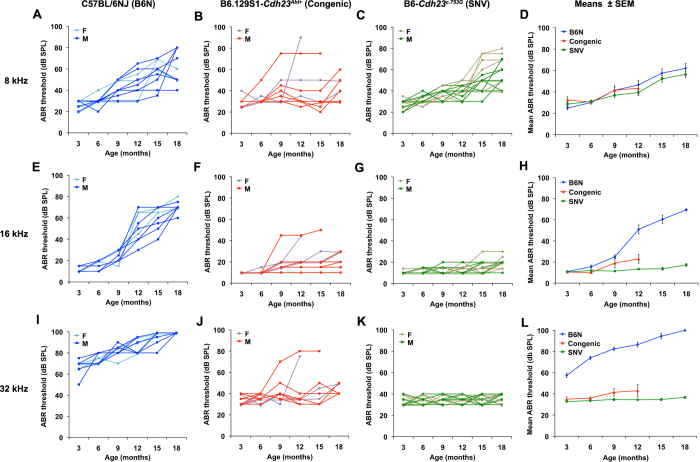
Progression of hearing loss in B6-*Cdh23*^*c.753G*^ SNV mice compared with congenic and inbred strain mice. Progression of hearing loss was assessed in C57BL/6 NJ (B6N) inbred strain mice (3 females and 7 males; **A,E,I**), B6.129S1-*Cdh23*^*Ahl*+^ (Congenic) strain mice (3 females and 7 males; **B,F,J**), and B6-C*dh23*^*c.753G*^ (SNV) strain mice (9 females and 9 males; **C,G,K**). ABR thresholds of individual mice tested at 3, 6, 9, 12, 15, and 18 months of age are shown for 8 kHz (**A–C**), 16 kHz (**E–G**), and 32 kHz (**I–K**) pure-tone stimuli, with sexes (F, M) of mice in each strain indicated by slightly different colors. Average ABR thresholds for each strain are shown for 8 kHz (**D**), 16 kHz (**H**), and 32 kHz (**L**) stimuli; vertical bars indicate standard errors of the means. Two congenic mice with exceptionally high thresholds (**B,F,J**) died before completion of the study and were excluded from the analysis; therefore, threshold averages for the congenic strain mice at the 15- and 18-month test ages are not presented (**D,H,L**).

Hearing was assessed by ABR threshold analysis. The high frequency 16 kHz and 32 kHz thresholds of B6-*Cdh23*^*c.753G*^ SNV mice remained at normal levels throughout the 18 month duration of the study (Fig. 3G,K) and were much lower than those of B6N controls (Fig. 3H,L), indicating a strong influence of the *Cdh23*^*c.753G*^ allele in preventing the progression of high frequency hearing loss. The 32 kHz threshold means of B6N mice were statistically significantly higher than those of SNV mice by 3 months of age, and the 16 kHz threshold means were significantly higher by 9 months of age (Table 2). The *Cdh23*^*c.753G*^ variant, however, had little effect on slowing the progression of lower frequency hearing loss, as shown by the 8 kHz thresholds of B6N and B6-*Cdh23*^*c.753G*^ SNV mice (Fig. 3D), which were not statistically significantly different at any test age (Table 2).

Although a reduction in high frequency hearing loss also was observed in the B6.129S1-*Cdh23*^*Ahl*+^ congenic strain mice (which also have the *Cdh23*^*c.753G*^ allele) compared with parental B6N mice (Fig. 3H,L), two of the 10 congenic mice tested exhibited rapid threshold increases between 6 and 12 months of age and died before the final 18 month test age (Fig. 3B,F,J), phenotypes not seen in B6-*Cdh23*^*c.753G*^ SNV mice.

### Hearing loss progression differs in 129S1, 129S1.B6-*Cdh23* ^*ahl*^, and 129S-*Cdh23* ^*c.753A*^ mice

**Figure 4:**
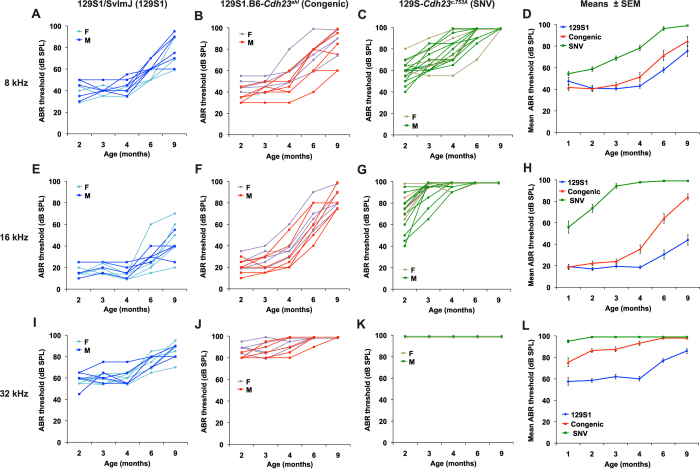
Progression of hearing loss in 129S-*Cdh23*^*c.753A*^ SNV mice compared with congenic and inbred strain mice. Progression of hearing loss was assessed in 129 S1/SvImJ (129S1) strain mice (6 females and 4 males; **A,E,I**), 129S1.B6-*Cdh23*^*ahl*^ (Congenic) strain mice (5 females and 6 males; **B,F,J**), and 129S-C*dh23*^*c.753A*^ (SNV) strain mice (7 females and 13 males; **C,G,K**). ABR thresholds of individual mice tested at 2, 3, 4, 6, and 9 months of age are shown for 8 kHz (**A–C**), 16 kHz (**E–G**), and 32 kHz (**I–K**) pure-tone stimuli, with sexes (**F,M**) of mice in each strain indicated by slightly different colors. Average ABR thresholds for each strain are shown for 8 kHz (**D**), 16 kHz (**H**), and 32 kHz (**L**) stimuli; vertical bars indicate standard errors of the means. Average ABR threshold values for the 1-month test age were obtained from additional mice not included in the longitudinal study: 6 males of the 129S1 strain, 4 females and 2 males of the Congenic strain, and 6 females and 4 males of the SNV strain.

Hearing loss progression was more rapid in both 129S1.B6-*Cdh23*^*ahl*^ congenic and 129S-*Cdh23*^*c.753A*^ SNV mice than in parental 129S1 mice (Fig. 4), indicating a deleterious effect of the *Cdh23*^*c.753A*^ variant. Although both strains share the same *Cdh23*^*c.753A*^ variant and the same 129S1 strain background, the progression of hearing loss in the 129S-*Cdh23*^*c.753A*^ SNV strain mice was more severe than that in 129S1.B6-*Cdh23*^*ahl*^ congenic mice. Already at one month of age, ABR thresholds of 129S-*Cdh23*^*c.753A*^ SNV mice were statistically significantly higher than those of 129S1.B6-*Cdh23*^*ahl*^ congenic mice for all test frequencies (Table 3).The differences in hearing loss progression between these strains suggest the influence of another locus (or loci) in the B6-derived congenic region of 129S1.B6-*Cdh23*^*ahl*^ that modifies the hearing loss effect of the *Cdh23*^*c.753A*^ variant in these mice.

### Cochlear hair cell loss corresponds with hearing loss

**Figure 5:**
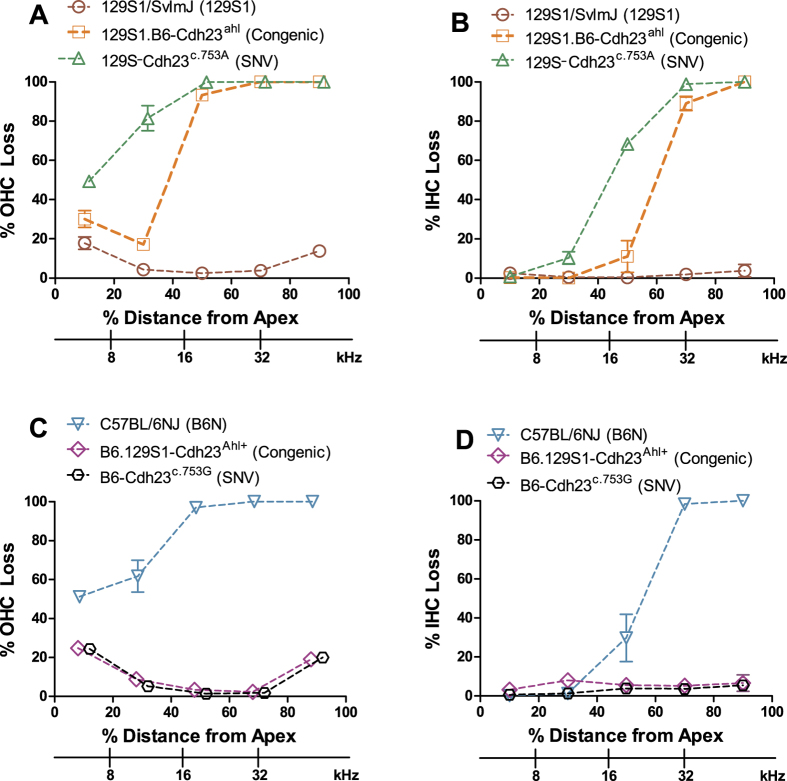
Cochlear hair cell loss associated with *Cdh23*^*c.753*^ variants. Cochleograms show the mean (+/−SEM, n = 4 cochlea per group) percent outer hair cell loss (OHC; **A,C**) and inner hair cell loss (IHC; **B,D**) in 20% intervals plotted as a function of percent distance from the apex of the cochlea. Cochlear locations related to frequency^36^ are shown on the x-axis. (**A,B**) Increased hair cell loss in 6-month-old 129S-*Cdh23*^*c.753A*^ (SNV) and 9-month-old 129S1.B6-*Cdh23*^*ahl*^(Congenic) mice compared with 9-month-old 129S1/SvImJ (129S1) parental strain mice indicates a strong influence of the *Cdh23*^*c.753A*^ allele in accelerating age-related hair cell degeneration. (**C,D**) Minimal hair cell loss in 18-month-old B6-*Cdh23*^*c.753G*^ (SNV) and B6.129S1-*Cdh23*^*Ahl*+^ (Congenic) mice compared with age-matched C75BL/6 NJ (B6N) parental strain indicates a strong influence of the *Cdh23*^*c.753G*^ allele in preventing age-related hair cell degeneration.

Consistent with the ABR results, there was minimal OHC loss and negligible IHC loss in B6-*Cdh23*^*c.753G*^ SNV mice and B6.129S1-*Cdh23*^*Ahl*+^ congenic mice at 18 months of age (Fig. 5C,D, n = 4/group; mean +/−SEM). In contrast, there were large OHC and IHC lesions in age-matched C57BL/6NJ mice (Fig. 5C,D, n = 4; mean +/−SEM; Supplementary Fig. S1). These results suggest a strong influence of the *Cdh23*^*c.753G*^ allele in preventing the age-related progression of OHC and IHC losses in the basal high-frequency region of the cochlea. In C57BL/6NJ mice, OHC losses were greater than the IHC loss and the lesions were greater in the base than the apex of the cochlea (Fig. 5C,D).

Consistent with ABR thresholds, mean (+/−SEM, n = 4/group) OHC and IHC losses in 9-month-old 129S1.B6-*Cdh23*^*ahl*^ congenic mice and 6-month-old 129S-*Cdh23*^*c.753A*^ SNV mice were much greater than in 9-month-old mice of the parental 129S1/SvlmJ strain (Fig. 5A,B; Supplementary Fig. S1) indicating the deleterious effect of the *Cdh23*^*c.753A*^ variant. Even though both strains share the same *Cdh23*^*c.753A*^ variant and the same 129S1 strain background, the degree of OHC and IHC loss in the 6-month-old 129S-*Cdh23*^*c.753A*^ SNV mice was more severe than the hair cell lesions seen in the 9-month-old 129S1.B6-*Cdh23*^*ahl*^ congenic mice. Hair cell lesions in the 129S1.B6-*Cdh23*^*ahl*^ congenic mice and 129S-*Cdh23*^*c.753A*^ SNV mice were more severe in the high-frequency basal region than the low-frequency apex, and OHC lesions were greater than IHC lesions at all cochlear locations. In contrast, OHC and IHC lesions in mice of the parental 129S1/SvlmJ strain were negligible.

### A linked locus on Chr 10 (*Mahl*) modifies *Cdh23* ^*c.753A*^ (*ahl)*-related hearing loss

**Figure 6:**
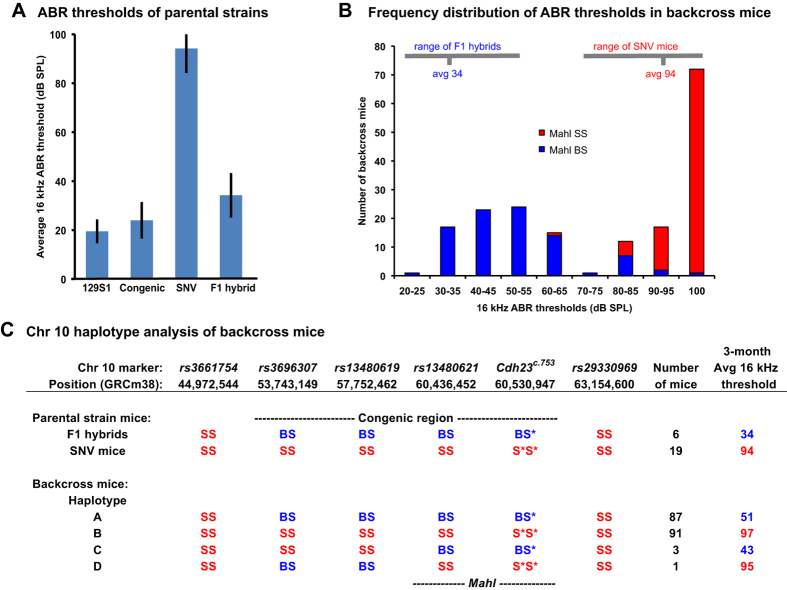
Localization of a hearing loss modifier in the 129S1.B6-*Cdh23*^*ahl*^ congenic region. (**A**) Average 16 kHz ABR thresholds (±standard deviations) of 3-month-old 129 S1 inbred strain mice (N = 10), 129S1.B6-*Cdh23*^*ahl*^congenic mice (Congenic; N = 10), 129S-*Cdh23*^c.753A^ mice (SNV; N = 19), and (Congenic × SNV) F1 hybrids (N = 6). (**B**) Frequency distribution of 16 kHz ABR thresholds among 3-month-old N2 mice (N = 182) from a backcross of (129S-*Cdh23*^c.753A^ SNV × 129S1.B6-*Cdh23*^*ahl*^ congenic) F1 hybrids with 129S-*Cdh23*^c.753A^ SNV mice. The threshold ranges and averages of age-matched parental strain mice (F1 hybrids, SNV mice) are indicated at the top of the figure as horizontal gray lines. N2 mice with homozygous and heterozygous *Mahl* genotypes are distinguished by red and blue colors. (**C**) Chr 10 haplotype analysis of N2 mice. SNP markers and their Mb positions along Chr 10 are shown above the corresponding genotypes of parental strain and N2 backcross mice. Haplotypes C and D contain crossovers within the congenic region, and the correlations of ABR thresholds with SNP genotypes further refine the candidate region of the modifier locus (designated *Mahl*). The 129S1.B6-*Cdh23*^*ahl*^congenic region and the *Mahl* candidate gene region are indicated by dotted lines. The non-recombinant *rs13480621* and C*dh23*^*c.753A*^ markers were used to assign *Mahl* genotypes in panel B above. Allele designations: B, C57BL/6-derived; S, 129S1-derived; S*, 129S1-derived with targeted *Cdh23*^*c.753G*>*A*^ substitution.

To genetically investigate the different rates of hearing loss progression in 129S1.B6-*Cdh23*^*ahl*^ congenic and 129S-*Cdh23*^*c.753A*^ mice, we produced F1 hybrids between these two strains and measured their ABR thresholds at three months of age, when ABR thresholds differ significantly between the parental strains (Fig. 6A). The F1 hybrid mice are homozygous for the *Cdh23*^*c.753A*^allele, heterozygous for 129S1 and B6 alleles at other loci within the congenic region, and homozygous for 129S1 alleles everywhere else in the genome. The thresholds of F1 hybrid mice were similar to those of 129S1.B6-*Cdh23*^*ahl*^ congenic mice and much lower than those of 129S-*Cdh23*^*c.753A*^ SNV mice (Fig. 6A), indicating a dominant effect of a postulated B6-derived, AHL-protective allele at a locus within the congenic region. To reveal the phenotype of the corresponding recessive 129S1-derived, AHL-promoting allele and reduce genetic complexity for mapping, the F1 hybrids were backcrossed to 129S-*Cdh23*^*c.753A*^ SNV mice rather than intercrossed. The ABR thresholds of the resulting N2 backcross progeny showed a strongly bimodal frequency distribution (Fig. 6B), indicating the strong influence of a single locus. If a single locus is primarily responsible for a trait, then only two genotypes affecting the trait will segregate in a backcross, resulting in a bimodal distribution. If multiple loci are responsible for a trait, there will be additional contributing genotypes, resulting in a more bell-shaped phenotypic distribution.

Allelic segregation analysis identified a locus within the Chr 10 congenic region of 129S1.B6-*Cdh23*^*ahl*^ that strongly associated with the hearing loss variation observed in the N2 progeny. We designated this locus *Mahl* for its modifying effect on the hearing loss of 129S1 mice homozygous for the *Cdh23*^*c.753A*^(*ahl*) variant. A total of 182 N2 generation progeny were produced from the backcross, and each mouse was assessed for ABR thresholds at three months of age and genotyped for 13 SNPs that differ between B6- and 129S1-strain DNA and that span the 20–70 Mb region of Chr 10. SNP marker genotypes were used to assign haplotypes to individual N2 mice to better define the 129S1.B6-*Cdh23*^*ahl*^ congenic region and to identify informative crossover events for narrowing the *Mahl* candidate interval. The strain of origin for the *Cdh23*^*c.753A*^ allele was distinguished by the presence or absence of the 104 bp PGK-Neo insertion remnant (Fig. 2B), which was inherited from the 129S-*Cdh23*^*c.753A*^ SNV strain. The 87 non-recombinant N2 mice that inherited B6-derived alleles from the F1 hybrid parent (haplotype A, Fig. 6C) exhibited low ABR thresholds (average 51 dB SPL), and the 91 non-recombinant N2 mice that inherited 129S1-derived alleles (haplotype B, Fig. 6C) exhibited high thresholds (average 97 dB SPL). Four N2 mice had recombinant chromosomes with informative crossovers in the Chr 10 congenic region. Analysis of these recombinant mice (haplotypes C and D, Fig. 6C) and their associated ABR thresholds reduced the candidate interval for *Mahl* to an approximately 5 Mb region (Fig. 6C).

Genotypes at the *Mahl* locus could account for about 80% of the total 16 kHz ABR threshold variation that was observed in the N2 backcross mice (Fig. 6B). Because total variation also includes environmental and experimental variation, the *Mahl* locus by itself may account for all of the underlying genetic variation. The amount of the total ABR threshold variance that could be explained by the *Mahl* locus was calculated as the difference between the total variance and the residual variance, expressed as a percent of the total variance. The total variance was calculated as the sum of squares for ABR thresholds of all 182 backcross mice, and the residual variance was calculated as the sum of squares for ABR thresholds of the 90 mice with the *Mahl* BS genotype plus the sum of squares for ABR thresholds of the 92 mice with the *Mahl* SS genotype.

## Discussion

Our results using homologous recombination in ES cells to produce single base pair substitutions in *Cdh23* add to the evidence from recent *in vivo* CRISPR-Cas9 gene editing studies^16^,^17^ showing that *Cdh23*^*c.753A*^ is the causative variant responsible for the age-related hearing loss effects ascribed to the Chr 10 *ahl* locus. C57BL/6 N mice with a CRISPR/Cas9-generated *Cdh23*^*c.753A*>*G*^ substitution were shown to retain normal ABR thresholds up to the final test age of 36 week^16^. The B6-*Cdh23*^*c.753G*^ SNV mice we generated also retain normal ABR thresholds at old ages, indicating that the loxP site retained after PGK-Neo excision (Fig. 1D) does not affect the phenotype of these mice. Our ABR (Fig. 3H,L) and cochleogram (Fig. 5C,D) results extend the time frame for high frequency (16 and 32 kHz) hearing loss protection conferred by the *Cdh23*^*c.753A*>*G*^ substitution in B6 mice to 78 weeks (18 months). The *Cdh23*^*c.753G*^ allele, however, did not fully prevent progression of lower frequency (8 kHz) hearing loss (Fig. 3D) and apical OHC loss (Fig. 5C) in B6 mice. A similar attenuation of high frequency but not low frequency hearing loss was reported in threshold comparisons between B6.CAST-*Cdh23*^*Ahl*+^ congenic mice and B6 mice^6^ and in comparisons between B6.CBA-*Cdh23*^*Ahl*+^ congenic mice and B6 mice^15^. Histological evidence for a frequency specific protective effect of the *Cdh23*^*c.753G*^ allele was also provided by the Kane *et al*.^15^ study, which showed that the degree of OHC loss in 18-month-old B6.CBA-*Cdh23*^*Ahl*+^ congenic mice is similar to that of age matched C57BL/6 J mice in cochlear regions corresponding to low frequencies (8 kHz and lower) but is much reduced in more basal regions corresponding to higher frequencies. Taken together, the results of these studies suggest that genetic factors other than the *Cdh23*^*c.753A*^ variant are responsible for the less commonly recognized low frequency hearing loss exhibited by B6 mice.

*Cdh23* knockout mice exhibit defects in hair bundle development and are congenitally deaf, whereas mice with a missense mutation of *Cdh23* exhibit normal hair bundle development but have defective tip links and exhibit a progressive hearing loss^18^, supporting the concept that non-severe *Cdh23* mutations may cause age-related hearing loss. The *Cdh23*^*c.753A*^ variant disrupts a splice donor site and is predicted to cause in-frame skipping of an exon encoding part of the 2nd and 3rd ectodomains of CDH23^14^. Because CDH23 is an integral component of stereocilia tip links^19^, the defect caused by exon skipping likely increases tip link breakage and slows repair mechanisms, which eventually could lead to cellular stress and hair cell apoptosis^20^. Our results are consistent with results from other studies^14^,^17^ showing that the *Cdh23*^c.753A^ splice site variant causes exon skipping (Fig. 2C). In our study, the estimated degree of exon skipping in mice with the *Cdh23*^c.753A^ variant appeared to correspond with the extent of high frequency hearing loss. 129S-*Cdh23*^*c.753A*^ SNV mice, which exhibit the earliest onset of 16 kHz hearing loss (<2 months), had the smallest estimated proportion of wild-type C*dh23* transcripts (18%) followed by 129S1.B6-*Cdh23*^*ahl*^ congenic mice (onset ~ 4 months, 24% wild-type transcripts) and B6N mice (onset ~ 9 months, 45% wild-type transcripts). These results suggest that genetic factors that modify the fidelity of *Cdh23* exon splicing may contribute to some of the hearing loss variability associated with the *Cdh23*^*c.753A*^ variant in different inbred mouse strains.

The *Cdh23*^*c.753A*^ variant (*ahl*) not only influences the progression of hearing loss in many inbred mouse strains^5^ but also has been shown to affect noise-induced hearing loss^21^ and to modify the hearing phenotypes of other gene mutations, including *Atp2b2*^*dfw*^ 22, *Adgrv1*^*frings*^ 23, *Sod1*^*tm1Leb*^ 24, *Myo7a*^*sh1-8J*^ 25, *Actb*^*tm1.1Erv*^ 26 and *Ush1g*^*js*^ 17. Thus an important advance of this study is the development of the B6-*Cdh23*^*c.753G*^ strain, which can be used to eliminate the potentially confounding effects of the *Cdh23*^*c.753A*^ variant when assessing other auditory phenotypes of mutations produced on the commonly used C57BL/6 J and C57BL/6 N sub-strains. To accomplish this, B6 mice carrying the mutation to be studied would first be crossed with B6-*Cdh23*^*c.753G*^ mice. The resulting F1 hybrid progeny (*Cdh23*^*c.753A/G*^) that have inherited the mutation would then be selected and backcrossed to mice of the B6-*Cdh23*^*c.753G*^strain. Finally, N2 progeny that have inherited the mutation and are homozygous for the *Cdh23*^*c.753G*^ allele would be interbred to establish a new mutant line for phenotype evaluation. An advantage of using the B6-*Cdh23*^*c.753G*^ strain described here is the ability to easily identify the targeted *Cdh23*^*c.753G*^ allele by PCR genotyping of size differences due to the closely linked 104 bp remnant of the PGK-Neo selection cassette (Figs 1D and 2B).

Although they have the same *Cdh23*^*c.753A/G*^substitutions and are on the same inbred strain backgrounds, congenic and SNV strain mice showed differences in their auditory phenotypes (Figs 3, 4, 5) that must be due to the effects of genetic factors located in the introgressed regions of the congenic strains. Three of the ten B6.129S1-*Cdh23*^*Ahl*+^ congenic strain mice examined exhibited decreased longevity and elevated ABR thresholds compared with B6-*Cdh23*^*c.753G*^ SNV mice (Fig. 3), suggesting that genetic incompatibilities may exist between the 129S1-derived congenic region and the B6 host genome in the B6.129S1-*Cdh23*^*Ahl*+^ mice. Because of the possible complications of co-transferred linked genes in congenic strains, genetically engineered mice with specific base pair changes are preferred for evaluating gene-specific phenotypic effects.

129S1.B6-*Cdh23*^*ahl*^ congenic strain mice and 12S1-*Cdh23*^*c.753A*^ SNV mice exhibited large differences in ABR thresholds (Fig. 4) and hair cell loss (Fig. 5). We used the differences in ABR thresholds of backcross mice derived from these two strains to genetically map a locus that modifies the hearing loss effect of the *Cdh23*^*c.753A*^ variant. We refined the map position of this locus, which we designate *Mahl*, to a 5 Mb interval within the 129S1.B6-*Cdh23*^*ahl*^ congenic region (Fig. 6C) and hypothesize that the 129S1 allele at this locus worsens *Cdh23*^*c.753A*^-related hearing loss relative to the B6 allele’s effect. The combinations of *Cdh23* and *Mahl* alleles can help explain the hearing loss differences observed among the 129S1 parental strain, 129S1.B6-*Cdh23*^*ahl*^ congenic strain, and 129S-*Cdh23*^*c.753A*^ SNV strain mice examined in this study (Supplementary Fig. S2).

On the basis of map position and inbred strain ancestry, we suspect that the *Mahl* locus may be equivalent to the previously described *Phl2* locus, which contributes to the progressive hearing loss of 101/H strain mice^27^. *Phl2* was mapped to the 51–78 Mb region of Chr 10, which overlaps with the 45–63 Mb congenic region of the 129S1.B6-*Cdh23*^*ahl*^ strain. The strains involved in the *Phl2* mapping crosses (101/H, MAI/Pas, and MBT/Pas) all have the *Cdh23*^*c.753A*^ allele, thereby excluding this variant as the underlying cause of the *Phl2-*related hearing loss. The 101/H strain and the 129S1 strain were derived from a recent ancestor^28^ and therefore may share the same deleterious allele at the *Phl2* locus. If so, the deleterious 129S1 allele at this locus (*Phl2/Mahl*) would be present in 129S-*Cdh23*^c.753A^ SNV strain mice (which exhibit high ABR thresholds), but replaced by the non-deleterious B6J allele in 129S1.B6-*Cdh23*^*ahl*^ congenic strain mice (which exhibit lower ABR thresholds).

We narrowed the candidate region for *Mahl* to the 58–63 Mb position on Chr 10, which includes the *Cdh23* gene. A comparative search of whole genome sequences of mouse inbred strains (http://www.sanger.ac.uk/science/data/mouse-genomes-project) did not reveal any sequence differences between C57BL/6 J (or C57BL/6NJ) and 129S1/SvImJ DNA that would affect splicing or cause amino acid changes in any of the 65 protein-coding genes located in the ~5 Mb *Mahl* candidate region. These results suggest that DNA differences in non-coding sequences or regulatory elements may be responsible for the modifier effect of the *Mahl* locus on hearing loss caused by the *Cdh23*^*c.753A*^ variant. Non-coding DNA differences within or surrounding the *Cdh23* gene itself cannot be ruled out, including those that may influence *Cdh23* expression levels or exon splicing fidelity.

The *Cdh23*^c.753A^ variant in the context of the 129S strain background (129S-*Cdh23*^c.753A^ SNV or 129S1.B6-*Cdh23*^*ahl*^ congenic mice) accelerates the progression of hearing loss (Fig. 4). In contrast, the presence of the same *Cdh23*^c.753A^ allele in CBA/CaJ mice (CBA.B6-*Cdh23*^*ahl*^ congenic mice) has no effect on hearing thresholds^15^. The effect that the *Cdh23*^*c.753A*^ variant has on hearing loss thus can vary greatly depending on strain background, having little or no effect on the CBA background^15^, a moderate effect on the B6 background (Fig. 3A,E,F), and a large effect on the 129S background (Fig. 4C,G,K). The strong effect of strain background on the severity of *Cdh23*^*c.753A*^-related hearing loss has potential implications for genetic studies of human age-related hearing loss. Specific screens of *CDH23* mutations in human subjects have identified variants that are weakly associated with both age-related^29^ and noise-induced^30^ hearing loss, but genome-wide association studies (GWAS) have failed to find any associations. Like the case for the *Cdh23*^*c.753A*^ variant in mice, the phenotypic expression of hypomorphic *CDH23* mutations in human populations may depend on the diverse genetic backgrounds of individuals, and the resulting decreased phenotypic penetrance would make associations with age-related hearing loss difficult to detect by GWAS.

## Methods

### Mice

All mice examined in this study originated from The Jackson Laboratory (http://www.jax.org/), and all procedures involving their use were performed at The Jackson Laboratory and approved by the Institutional Animal Care and Use Committee. All methods used in the study were performed in accordance with the guidelines and regulations of the U.S. National Institutes of Health (NIH) Office of Laboratory Animal Welfare (OLAW) and the Public Health Service (PHS) Policy on the Humane Care and Use of Laboratory Animals. The Jackson Laboratory is accredited by the American Association for the Accreditation of Laboratory Animal Care.

### Congenic strain development

The B6.129S1-*Cdh23*^*Ahl*+^ and 129S1.B6-*Cdh23*^*ahl*^ reciprocal congenic strains were developed by genetic introgression of B6J- and 129S1-derived segments of Chromosome 10 (containing the *Cdh23* gene) into the genomes of 129S1 and B6J, respectively. To accomplish this introgression, mice from the B6J and 129S1 parental strains were first intercrossed to generate F1 hybrids. The F1 hybrids were then backcrossed to B6J mice to produce N2 generation mice for development of the B6.129S1-*Cdh23*^*Ahl*+^ strain and to 129S1 mice to produce N2 mice for development of the 129S1.B6-*Cdh23*^*ahl*^ strain. Repeated backcrossing of hybrid progeny (selected on the basis of Chromosome 10 marker genotypes) to the parental (B6J or 129S1) strain continued until 10 backcross generations (N10) were completed. Finally, the N10 generation mice of each line were sibling inbred to produce the two homozygous congenic strains. To define the extent of the congenic regions in each strain, single nucleotide polymorphisms (SNPs) along the length of Chromosome 10 were genotyped.

### Genetic engineering of B6-*Cdh23* ^*c.753G*^ and 129S-*Cdh23* ^*c.753A*^ mice

Single base pair substitutions in the *Cdh23* gene of C57BL/6 (B6) and 129S1/SvImJ (129S1) strain mice were produced using a combination of recombineering^31^,^32^ and standard molecular cloning techniques. Briefly, a 9,728 bp fragment (GRCm38 assembly, Chr10: 60,527,162–60,536,889) of the *Cdh23* gene containing the targeted nucleotide (c.753) was retrieved from bacterial artificial chromosome (BAC) clones and integrated into embryonic stem (ES) cells by homologous recombination (Fig. 1). To produce B6-*Cdh23*^*c.753G*^knock-in mice, the *Cdh23* fragment from a 129S1 BAC clone was incorporated into the genome of B6N ES cells (JM8.A3), and to produce 129S-*Cdh23*^*c.753A*^ knock-in mice, the *Cdh23* fragment from a B6 BAC clone was incorporated into the genome of 129S1-derived ES cells (ES-CJ7). The *Cdh23*^*c.753A/G*^ single nucleotide polymorphism (SNP) is the only DNA sequence difference between B6 and 129S1 in the entire targeted region (9,728 bp), and the natural strain variants precluded the need to create mutations in the targeting vectors.

To create the targeting vector, a synthetic gene (Genscript, Piscataway, NJ) consisting of a 300 bp homology arm (Chr10: 60,536,591–60,536,889) and a 320 bp homology arm (Chr10: 60,527,162–60,527,481) and including restriction enzyme sites (*Hind*III and *Xho*1, spaced by *Kpn*1) for retrieval and *Not*1 for construct release was cloned into the pBlight-TK plasmid^33^. This plasmid then was used to retrieve the targeted *Cdh23* fragments from BAC DNA by gap repair in competent bacteria, and DNA was sequenced to confirm the presence of the targeted nucleotide. A PGK-Neo cassette with flanking LoxP sites and homology arms was synthesized and inserted by recombineering into the *Cdh23* fragment of the targeting vector, 178 bp downstream of the targeted c.753 nucleotide. The final plasmid vector was confirmed by DNA sequence analysis, linearized with *Not*I and electroporated into ES cells. ES cells under G418 positive selection were screened by a loss of allele assay against the PGK-Neo insertion site, and positive ES cell clones were confirmed by Southern blot and DNA sequence analysis.

Verified B6N JM8A3 ES cell clones were microinjected into 3.5 dpc blastocyst-stage albino C57BL/6 J host embryos. After surgical transfer of the microinjected embryos and development to term, chimeras were identified by coat color and high chimera males were mated with C57BL/6NJ females. Progeny were screened for the presence of PGK-Neo and the targeted *Cdh23*^*c.753G*^ substitution. The floxed PGK-Neo cassette then was deleted by mating with B6N.Cg-Tg(Sox2-cre) 1Amc/J mice (Stock #14094). Mice heterozygous for the *Cdh23*^*c.*753G^ substitution, with the PGK-Neo cassette excised and lacking the Cre transgene, were mated to C57BL/6NJ mice, and progeny heterozygous for the *Cdh23*^*c.753G*^ substitution then were intercrossed to establish a homozygous B6-*Cdh23*^*c.753G*^ line, confirmed by DNA sequence analysis (Fig. 2A). The formal name for this strain is B6N(Cg)-*Cdh23*^*tm2.1Kjn*^/Kjn (Jackson Laboratory Stock #18399).

Verified 129S1/SvImJ ES cell clones were microinjected into 3.5 dpc blastocyst-stage non-agouti B6 host embryos. After surgical transfer of the microinjected embryos and development to term, chimeras were identified by coat color, and high chimera males were mated with 129S1/SvImJ females. Progeny were screened for the presence of PGK-Neo and the closely linked targeted *Cdh23*^*c.753A*^ substitution. The floxed PGK-Neo cassette then was deleted by mating with 129S/Sv-Tg(Prm-cre)58Og/J mice (Stock #3328). Mice heterozygous for the *Cdh23*^*c.753A*^ substitution and lacking the PGK-Neo cassette and Cre transgene were mated to 129S1/SvImJ mice, and progeny heterozygous for the *Cdh23*^*c.753A*^ allele then were intercrossed to establish the homozygous 129S-*Cdh23*^*c.753A*^strain, confirmed by DNA sequence analysis (Fig. 2A). The formal name for this strain is 129S/Sv-*Cdh23*^*tm1.1Kjn*^/Kjn (Stock #16208).

### PCR genotyping method to identify genetically engineered *Cdh23* ^*c.753A/G*^ substitutions

A simple PCR-based genotyping method was developed to distinguish wild type alleles from those with genetically engineered single base pair substitutions (Fig. 2B). The forward primer CTTAAGCTCGGCCAAACATC and the reverse primer CACAACAGGAAGAAAGCAAGC, which flank the site of the excised PGK-Neo selection cassette, were used to amplify PCR products that distinguish alleles based on the presence or absence of the post-excision 104 bp cassette remnant, which serves as a convenient marker for the targeted *Cdh23*^*c.753*^ SNVs.

### cDNA synthesis and RT- PCR analysis

For each of the six strains analyzed in this study, total RNA from eight cochleae (pooled from four 6-week-old male mice) was extracted and purified with the RNeasy Mini Kit (Cat. No. 74104, Qiagen, Valencia, CA) using the manufacturer’s procedures. Total RNA quantity and quality were determined using the Agilent 2100 Bioanalyzer (Agilent Technologies, Palo Alto, CA). First strand synthesis of cDNA was performed using the High Capacity cDNA Reverse Transcription Kit (Cat. No. 4368814, Thermo Fisher Scientific, Waltham, MA) in accordance with manufacturer’s instructions, but using a *Cdh23* gene-specific primer (RT-Cdh23R, AGACAATAGTGTAGCCAATCCCACGG) rather than random primers.

To evaluate alternative splicing, RT-PCR primers were designed from DNA sequences located in the *Cdh23* exons that flank the variably skipped exon: forward primer (RT-Cdh23F, ACACCAGTGGGGACACCCATCTTCATC) and reverse primer RT-Cdh23R (the same primer shown above for reverse transcription). The same RT-PCR primers were used in a previously reported assay of *Cdh23* exon skipping^17^. RT-PCR amplifications for each of the cDNA samples were performed using the MasterTaq Kit (Cat. No. 2200210, 5 PRIME, Inc., Gathersburg, MD) with HotStart-IT Taq DNA polymerase (Cat. No. 71195, Affymetrix, Santa Clara, CA), and run in a Bio-Rad Peltier Thermal Cycler. Amplification consisted of one cycle of denaturation at 97 °C for 30 seconds followed by 40 cycles, each consisting of 94 °C for 30 seconds, 58 °C for 30 seconds, and 72 °C for 30 plus 1 second per cycle. After the 40 cycles, the final product was extended for 10 minutes at 72 °C. PCR products were visualized on 2% agarose gels stained with ethidium bromide. The ImageJ software program (https://imagej.nih.gov/ij/) was used to process digital images of the gels and quantify the densities of gel bands corresponding to alternatively spliced transcripts.

### Auditory brainstem response (ABR) measurements to assess hearing loss

ABR thresholds were measured at 8, 16 and 32 kHz in a sound attenuating chamber using the SmartEP auditory evoked potential diagnostic system from Intelligent Hearing Systems (IHS, Miami, FL) as described previously^8^. Briefly, mice were anesthetized with tribromoethanol (0.2 ml of 20 mg/ml stock per 10 g of body weight, i.p.) and placed on a temperature controlled heating pad to maintain body temperature at 37 °C. Three subdermal electrodes, placed at the vertex and behind each ear, were used to record brain stem responses to defined tone-bursts (3 ms duration. 1.5 ms cosine-gated rise/fall time). The responses were then amplified, filtered (100–3000 Hz) and averaged (25 kHz sampling rate, 10 ms analysis window). Stimulus intensity was initially decreased in 10 dB steps until the response began to disappear and then lowered in 5 dB steps; ABR threshold was defined as the lowest intensity at which an ABR response could be reliably obtained. With our testing system, average ABR thresholds for normal hearing mice are about 40, 20, and 45 dB SPL for 8, 16 and 32 kHz stimuli, respectively. The same individual performed all of the ABR threshold determinations throughout the study and was uninformed of the ages, strains, and genotypes of mice at the time of the tests.

### Cochleograms to assess hair cell loss

Our procedures for preparing cochleograms have been described in detail in earlier publications^34^,^35^. Briefly, after completing the ABR measurements at The Jackson Laboratory, the mice were euthanized with CO_2_, decapitated, and their bullae quickly removed and opened to expose the inner ear. A small hole was carefully made in the round window and 10% formalin fixative was gently perfused through the opening. Afterward, the cochlea was immersed in fixative and shipped to University of Buffalo for analysis. Cochleae were stained with Ehrlich’s hematoxylin solution. A person, blind to the ABR results, dissected out the organ of Corti into two to three half turns. The samples were then mounted as a flat surface preparation in glycerin on glass slides, coverslipped and examined with a light microscope (Zeiss Standard, 400X). Inner hair cells (IHC) and outer hair cells (OHC) were counted along successive 0.12–0.24 mm intervals from the apex to the base of the cochlea by a second person blind to the experimental conditions. A hair cell was counted as present if both the cuticular plate and hair cell nucleus were clearly visible and considered missing if either were absent. Using lab norms and custom software, the percentages of missing IHC and OHC were determined for each animal and a cochleogram constructed showing the percentage of missing OHC and IHC as a function of percent distance from the apex of the cochlea. Position in the cochlea was related to frequency using a mouse tonotopic map^36^. Mean percent OHC loss and mean percent IHC loss were computed over 20% intervals of the cochlea for four cochleae in each of the six experimental groups, at 6 months of age for 129S-*Cdh23*^*c.7*53A^ mice, at 9 months of age for 129S1/SvlmJ and 129S1.B6-*Cdh23*^*ahl*^ mice, and at 18 months of age for C57BL/6NJ, B6.129S1-*Cdh23*^*Ahl*+^ and B6-*Cdh23*^*c.753G*^ mice. Cochlear surface preparations were photographed with a digital camera (SPOT Insight, Diagnostic Instruments Inc.) attached to a Zeiss Axioskop microscope, processed with imaging software (SPOT Software, version 4.6), Adobe Photoshop 5.5.

### Genotyping markers for genetic mapping

For genetic mapping analysis of the (129S-*Cdh23*^*c.753A*^ × 129.B6-*Cdh23*^*ahl*^) × 129S-*Cdh23*^*c.753A*^ backcross, tail-tip samples from 182 individual N2 progeny mice were provided to the Jackson Laboratory’s Genotyping Service, which outsources SNP genotyping to LGC Genomics (Beverly MA). SNP genotyping was performed for 13 selected SNPs on Chr 10 that differed between the B6 and 129S1 strains. For genotyping the *Cdh23*^*c.753*^ locus, 129S1- and B6-derived alleles were distinguished by the presence or absence of the of the 104 bp PGK-Neo insertion remnant (Fig. 2B).

### Statistical analysis

ABR data analysis was performed using the JMP 7.0 interactive statistics and graphics software program (www.JMP.com). Statistical significance of the differences among means was determined by one-way ANOVA with post-hoc Tukey HSD test for multiple pair-wise comparisons.

## Additional Information

**How to cite this article:** Johnson, K. R. *et al*. Effects of *Cdh23* single nucleotide substitutions on age-related hearing loss in C57BL/6 and 129S1/Sv mice and comparisons with congenic strains. *Sci. Rep.* **7**, 44450; doi: 10.1038/srep44450 (2017).

**Publisher's note:** Springer Nature remains neutral with regard to jurisdictional claims in published maps and institutional affiliations.

## Supplementary Material

Supplementary Information

## Figures and Tables

**Table 1:** Abbreviated inbred strain designations used throughout the paper, followed by the type of strain, the full strain name and the Jackson Laboratory stock number.

Abbreviation	Type	Full strain name	Stock #
B6J^a^	parental	C57BL/6 J	664
B6N^a^	parental	C57BL/6NJ	5304
129S1	parental	129S1/SvImJ	2448
B6.129S1-*Cdh23*^*Ahl*+^	congenic	B6.129S1-(*rs13480546-rs13480629*)/Kjn	21479
129S1.B6-*Cdh23*^*ahl*^	congenic	129S1.B6-(*rs3696307-rs257098870*)/Kjn	18926
B6-*Cdh23*^*c.753G*^	SNV^b^	B6N(Cg)-*Cdh23*^*tm2.1Kjn*^/Kjn	18399
129S-*Cdh23*^*c.753A*^	SNV^b^	129S/Sv-*Cdh23*^*tm1.1Kjn*^/Kjn	16208

^a^The designation C57BL/6 (or B6) is used when referring to either C57BL/6 J or C57BL/6 NJ.

^b^Genetically engineered strain with a single nucleotide variant (SNV).

**Table 2:** Statistical comparisons of ABR threshold means at different ages: C57BL/6NJ (B6N, N = 10), B6.129S1-*Cdh23* ^*Ahl*+^ (Congenic, N = 10), and B6-*Cdh23* ^*c.753A*^ (SNV, N = 18).

ABR thresholds			Tukey-Kramer HSD test P values					
	Pairwise Comparisons		3 months	6 months	9 months	12 months	15 months	18 months
8 kHz	B6N	Congenic	0.11	0.95	0.99	0.87	—	—
8 kHz	B6N	SNV	0.06	0.71	0.44	0.39	0.13	0.32
8 kHz	Congenic	SNV	0.99	0.90	0.44	0.73	—	—
16 kHz	B6N	Congenic	0.86	0.004	0.08	<0.0001	—	—
16 kHz	B6N	SNV	0.86	0.04	<0.0001	<0.0001	<0.0001	<0.0001
16 kHz	Congenic	SNV	0.99	0.35	0.008	0.03	—	—
32 kHz	B6N	Congenic	<0.0001	<0.0001	<0.0001	<0.0001	—	—
32 kHz	B6N	SNV	<0.0001	<0.0001	<0.0001	<0.0001	<0.0001	<0.0001
32 kHz	Congenic	SNV	0.46	0.33	0.06	0.10	—	—

**Table 3:** Statistical comparisons of ABR threshold means at different ages: 129S1/SvImJ (129S1, N = 10), 129S1.B6-*Cdh23* ^*ahl*^ (Congenic, N = 11), and 129S-*Cdh23* ^*c.753A*^ (SNV, N = 20).

ABR thresholds			Tukey-Kramer HSD test P values					
	Pairwise Comparisons		1 month	2 months	3 months	4 months	6 months	9 months
8 kHz	129S1	Congenic	0.12	0.99	0.6	0.23	0.02	0.15
8 kHz	129S1	SNV	0.04	<0.0001	<0.0001	<0.0001	<0.0001	<0.0001
8 kHz	Congenic	SNV	<0.0001	<0.0001	<0.0001	<0.0001	<0.0001	0.0016
16 kHz	129S1	Congenic	0.99	0.59	0.42	<0.0001	<0.0001	<0.0001
16 kHz	129S1	SNV	<0.0001	<0.0001	<0.0001	<0.0001	<0.0001	<0.0001
16 kHz	Congenic	SNV	<0.0001	<0.0001	<0.0001	<0.0001	<0.0001	<0.0001
32 kHz	129S1	Congenic	0.0007	<0.0001	<0.0001	<0.0001	<0.0001	<0.0001
32 kHz	129S1	SNV	<0.0001	<0.0001	<0.0001	<0.0001	<0.0001	<0.0001
32 kHz	Congenic	SNV	<0.0001	<0.0001	<0.0001	0.0014	0.71	1.0
