# Supplementary material for: Effects of Cdh23 single nucleotide substitutions on age-related hearing loss in C57BL/6 and 129S1/Sv mice and comparisons with congenic strains
Source: Sci Rep. 2017 Mar 13;7:44450. doi: 10.1038/srep44450 (PMC5347380; doi:10.1038/srep44450)

Effects of *Cdh23* single nucleotide substitutions on age-related hearing loss in C57BL/6 and 129S1/Sv mice and comparisons with congenic strains

Kenneth R. Johnson[1*], Cong Tian[1], Leona H. Gagnon[1], Haiyan Jiang[2], Dalian Ding[2], Richard Salvi[2]

[1] The Jackson Laboratory, Bar Harbor, Maine 04609, USA
[2] Center for Hearing and Deafness, University at Buffalo, Buffalo, NY 14214, USA

Supplementary Figure S1.

Cochlear photomicrographs showing extent of hair cell loss.
Representative photomicrographs showing the presence or absence (yellow star symbol) of inner hair cells (IHC) and outer hair cells (OHC) from the same location in the upper basal turn of the cochlea: (A) 9-month-old 129S1/SvImJ parental strain mouse, (B) 9-month-old 129S1.B6-*Cdh23*^{ahl} congenic mouse, (C) 6-month-old 129S-*Cdh23*$^{c.753A}$ SNV mouse, (D) 18-month-old B6-*Cdh23*$^{c.753G}$ SNV mouse, (E) 18-month-old B6.129S1-*Cdh23*$^{Ahl+}$ congenic mouse, (F) 18-month-old C57BL/6NJ parental strain mouse.

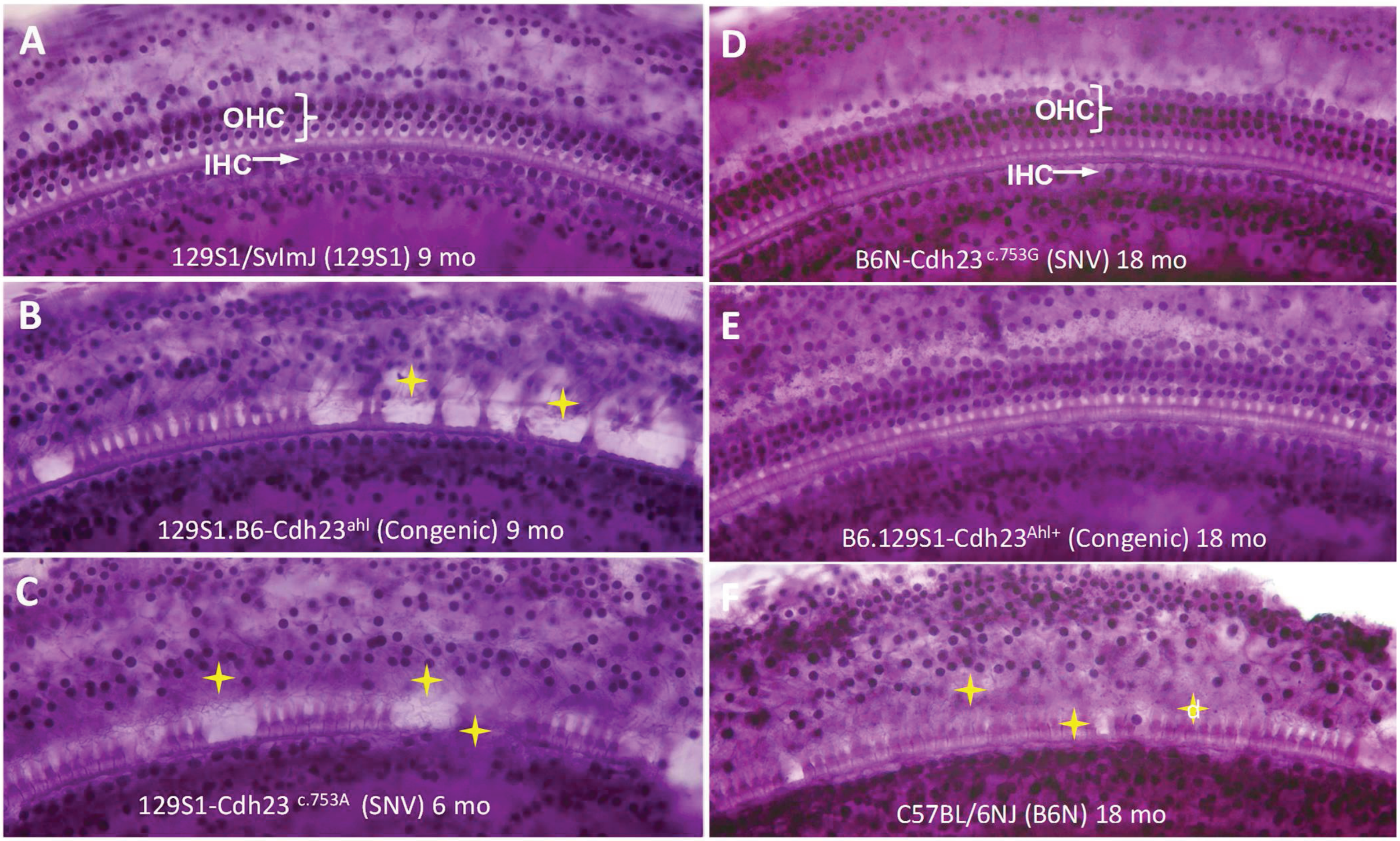

Supplementary Figure S2.

Chromosome 10 model to explain *Mahl* effects on hearing loss.
For each strain (129S1/SvImJ, 129S1.B6-*Cdh23*^{ahl}, and 129S-*Cdh23*$^{c.753A}$), the *Cdh23* and *Mahl* loci are shown diagrammatically as black or white boxes at their approximate Mb positions on Chr 10. White boxes represent alleles conferring AHL resistance, and black boxes represent alleles conferring AHL susceptibility. DNA derived from C57BL/6 is shown in blue, and DNA derived from 129S1 is shown in orange. Average 32 kHz ABR thresholds at 3 months of age are shown to the right of each strain's chromosome to show the hearing loss effects of the *Cdh23-Mahl* allelic combinations.

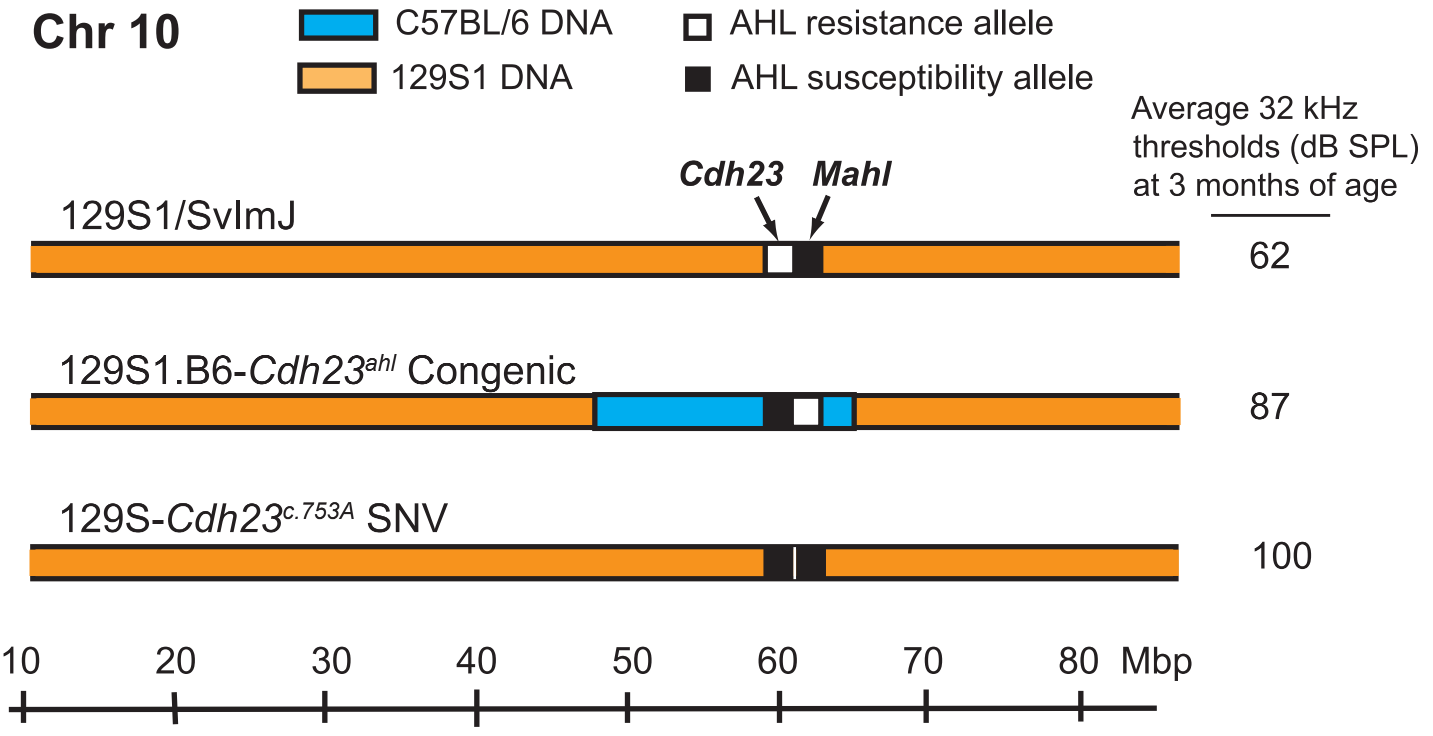

Supplement: Supplementary Information [file srep44450-s1.pdf]
